# A Corporate Wellness Program and Nursing Home Employees' Health

**DOI:** 10.3389/fpubh.2020.531116

**Published:** 2020-10-30

**Authors:** Gabriela Kernan, Manuel Cifuentes, Rebecca Gore, David Kriebel, Laura Punnett

**Affiliations:** ^1^Department of Public Health, University of Massachusetts Lowell, Lowell, MA, United States; ^2^Department of Public Health, Regis College, Weston, MA, United States; ^3^Department of Biomedical Engineering, University of Massachusetts Lowell, Lowell, MA, United States

**Keywords:** body mass index, leisure-time exercise, health behaviors, work environment, healthcare workers, nursing homes, interventions

## Abstract

**Background:** Many employed Americans suffer from chronic conditions like obesity, diabetes, and cardiovascular diseases. Worksite wellness programs provide opportunities to introduce health promotion strategies. While there is evidence of the effectiveness of workplace health promotion, this is tempered by concern that benefits may be less available to low-wage workers with inflexible working conditions.

**Objective:** The aim was to evaluate a workplace health promotion (WHP) in the long-term care sector (skilled nursing facilities).

**Methods:** Nursing home employees from 18 facilities within a single company were surveyed by a standardized, self-administered questionnaire. A company-sponsored WHP program was offered to the facilities, which were free to take it up or not. We categorized the facilities by level of program adoption. Cross-sectional associations were estimated between program category and prevalence of individual-level worker health indicators, adjusting for center-level working conditions.

**Results:** A total of 1,589 workers in 5 job categories completed the survey. Average levels of psychological demands and social support at work were relatively high. Supervisor support stood out as higher in centers with well-developed WHP programs, compared to centers with no programs. There were no differences among program levels for most health outcomes. Workers in centers with well-developed programs had slightly lower average body mass index and (unexpectedly) slightly lower prevalence of non-smoking and regular aerobic exercise.

**Conclusions:** Only small health benefits were observed from well-developed programs and working conditions did not appear to confound the negative results. This low-intensity, low-resourced workplace health promotion program may have benefited a few individuals but seems to have had only modest influence on average levels of the measured health indicators. Many nursing home employees experience obstacles to health behaviors; approaches that provide more environmental and economic supports for healthy behaviors, such as *Total Worker Health*®, may yield larger health benefits.

## Introduction

Most adults in the United States are employed and spend on average of 8.5 h per day in a work-related activity. Workplaces thus provide an environment to educate employees how to adopt healthy lifestyles (1). Worksite health promotion (WHP) has been recognized as a public health strategy (2) and a number of large US employers offer some type of wellness programming as a part of their employees' health benefits (3). At the same time, working conditions represent potential obstacles to health behaviors (4). The dilemma is under-studied in the literature.

WHP programs are typically intended to modify employee health behaviors in order to reduce risk for chronic health conditions. Typical components include some form of health assessment and education about smoking, alcohol consumption, healthy eating, sleep, and exercise (5–7). In the United States, the underlying premise of WHP is simple: A healthy workforce can be financially beneficial to the employer by lowering medical health care spending (8, 9). Employers who initiate such programs are typically motivated by goals such as decreasing absenteeism, increasing job satisfaction, and reducing the cost of group health care coverage (9–13). There is some evidence that healthier employees are more productive and are less likely to miss work (10, 14, 15). Over the past three decades, the popularity of WHP programs has increased notably (1, 15, 16).

However, there is no clear consensus with respect to empirical WHP program effectiveness and benefits (16–20). Rongen et al. (21) conducted a systematic review of randomized clinical trials of the effect of workplace health promotion programs on smoking cessation, physical activity, healthy nutrition, and/or obesity, self-perceived health, work absence due to sickness, work productivity, and concluded that overall effect of WHP programs are small. Studies that have reported positive WHP effectiveness are often not free of methodological issues (21–25). Some suffered from small sample size (19), while some larger, longitudinal studies found no or very little effect (5, 26).

Another important caveat concerns program setting and limits on generalizability. Many positive studies had participation mostly from better-off employees, with unequal proportions by race or ethnicity and barriers such as working conditions and low socioeconomic status unaccounted for (27, 28). Most published studies from the United States describe WHP programs that rely on group health insurance resources (16, 18, 29, 30). Low-wage workers often cannot afford health insurance offered by their employers and therefore do not have access to those programs (28). Many other countries organize their preventive health care and medical insurance differently, meaning that income is not a barrier to services; results from these studies would not necessarily apply to the U.S. context.

The lack of consistency in the research literature prompts us to a point where we must ask ourselves “Are WHPs working?” If so, is it true in all settings, or only in a few, highly selective ones? Moreover, are they equally effective for all workers? (31).

Worksite health promotion activities typically target health behavior choices by individuals but do not often consider the fact that behaviors tend to cluster in certain populations and are not randomly distributed among groups. For example, SES is negatively associated with BMI (32), which may reflect a wide range of mediating causal variables (4). Therefore, health promotion programs should consider the environmental and mediating factors that affect specific groups, whether defined by socioeconomic status, ethnicity, and/or occupation.

With the projection of the healthcare sector growing faster than others, this workforce's health is a necessity (33). Nursing aides employed in nursing homes are a vulnerable low-wage population; most of them are middle-aged, and many also are recent immigrants or single parents. They work long hours to make ends meet (32). Finding time and energy for exercise may be impossible after a physically or emotionally fatiguing workday; difficulty in balancing work with family demands, especially common for working women, may exacerbate this. Comfort eating, as well as other unhealthy behaviors, serves as coping strategies for many workers to better tolerate or relieve work-induced fatigue and/or stress (34). Shiftwork and excessively long work hours disrupt sleep and metabolism, in turn increasing the risk of obesity and metabolic syndrome. Night work also interferes with exercise through physiological as well as behavioral mechanisms (35, 36).

The overall objective of this study was to evaluate a workplace health promotion (WHP) program in a sample of long-term care facilities (nursing homes). We sought to compare facilities with different levels of WHP programming in terms of workers' health behaviors, and perceived working conditions. Specific research questions were: (1) Are there differences related to facility WHP programs with respect to employee health behaviors, health beliefs, or working conditions; and (2) does social support from coworkers and/or supervisors mediate the association between WHP and health outcomes.

## Materials and Methods

This cross-sectional study is based on a larger project [“Promoting Caregivers' Physical & Mental Health via Transdisciplinary Intervention (ProCare)”] examining health of employees of long-term nursing facilities located in several states of the U.S. and managed by one company (37–40). Each center was provided with educational materials for employees and (at first) a small annual budget for health promotion activities, which was subsequently canceled. The centers had the freedom to use it or not and to design their own WHP strategies. No release time was authorized for employees to participate in any activities. To our knowledge, there were no professional wellness consultancies contracted, and no WHP professionals were employed directly by the company.

The independent variable was WHP programming level, classified at the facility level. Data were collected by multiple methods. Two rounds of a survey were distributed to management representatives (facility director, Director of Nursing, etc.) to gather information on type of activities, their length and frequency, and who sponsored them within the center. Activities were counted by category targeting specific health behaviors: eating habits, weight management, voluntary exercise, stress reduction, and other health promotion topics (Table 1). This was supplemented by a brief survey distributed by the investigators, in an attempt to fill in gaps from non-respondents to the corporate surveys.

**Table 1:** Selected questions from corporate survey of center representatives about WHP activities offered.

**For the past year, indicate which of these activities were done, number of participants, length of time that the activity was in place, and number of times that it was offered**.
**Healthy eating (examples)**
“Healthy” vending machine foods; Healthy “light” recipe swap; Healthy potluck/bag lunch group; Healthy food tasters contest
**Weight loss (examples)**
Biggest Loser; Weight Watchers; Weight loss program discount
**Exercise (examples)**
Tai Chi, Yoga, Aerobics; Competition for walking miles, lost inches, etc.; Exercise room on site; Bicycle rack to parking lot; Allow exercise during work time; Designated walking route around center grounds; Walking club; Use of center's equipment
**Stress reduction (examples)**
Traveling massage; Quiet room; Meditation class; Relaxation techniques class
**Health promotion (examples)**
Blood pressure clinics; Health fair with screenings; Smoking cessation program; Wellness bulletin board or newsletter

For each of the 18 centers, a wellness composite score was developed based on the sum of activities and program classification. Some centers had formal wellness programs in place, while some had only informal employee-based initiatives. WHP programming was categorized as: (1) “well developed,” meaning there was a formal plan at the center with at least three different health activities, program champion, and/or committee; (2) “emerging,” meaning employee-initiated only, with one or two activities offered; (3) no WHP; or (4) unknown status (no response to any survey).

Data from individual workers were collected by self-administered questionnaires. The population comprised of active direct-care employees: nursing aides (CNAs, GMAs, etc.), licensed practical nurses (LPNs), and registered nurses (RNs). Other occupations such as office, clerical, janitorial, food, and recreational services were defined as not eligible. The procedures of survey administration were described in details elsewhere (37–39). The study was approved by the University of Massachusetts Lowell Institutional Review Board (IRB #06-1403).

Outcome variables were measured using validated instruments, when possible. Health behaviors were assessed by questions about the frequency of weekly physical exercise, smoking habits, and sleep quality. Self-reported height and weight were used to calculate body mass index (BMI). Self-rated health (mental and physical) were measured by the SF-12 (41). Behavioral changes within the last 3 months were assessed by a set of items using the same question stem: “Have you changed during the last 3 months how often you do any of the following:” (1) Eat high fat food, (2) Eat a diet high in fiber, (3) Try to lose weight, (4) Exercise, (5) Have stress in my life, (6) Drink alcohol, and (7) Get a full night sleep. The ratings varied from 1 (much less often than the participant used to do) to (5 much more often than used to do).

Measured working conditions included perception of supervisory and coworker support (2 items each), job strain defined as the ratio of psychological demand (effort required to perform the job and time pressure; 2 items) to decision-making latitude (combination of job decision-making authority and the opportunity to use and develop skills on the job; 2 items), physical job exertion (42), workers' perception of control over their work schedule (2 items) (43), perception of safety at work (4 items; 2 from Griffin and Neal (44) and 2 developed by investigators, workplace assault in the past 3 months measured by a single item: “Have you been kicked, grabbed, pushed or scratched by a patient, patient's visitor, or family member?” Beliefs about health were measured by 9 behavior-specific self-efficacy items, which are considered amenable to change following positive or negative experiences (44), and internal health locus of control, which is considered stable throughout adulthood (45).

The SAS® 9.2 system was used for data management and analysis. Cross-tabulation and ANOVA (fitted via PROC GLM) were utilized to compare differences across WHP program levels. Chi-square statistics were employed to test the cross-tabulation results. Schaffe test was used in conjunction with ANOVA to find the mean differences between groups (46). Statistical significance was based on alpha of 0.05.

Separate multivariate models were fitted to examine whether working conditions or health behaviors were associated with program status, starting with variables that differed (*p* < 0.05) in bivariate analyses. Generalized linear models were constructed using the Genmod procedure with link log and identity function. Centers with no WHP were used as a comparison group. Covariates included in the regression models were gender (male or female), job category (nursing aide or other), age, and place of residence (New England or Maryland), as these demonstrated variability across programs. Decision about retention of covariates in the multivariate models was based on the change-in-estimate criterion, keeping the variable in the final model if adding it changed the effect estimates of program status on outcome by 10% or more (47).

## Results

Two, four, and seven centers were classified as having well-developed, emerging, and no WHP programs, respectively. Five centers did not respond and were designated as unknown. Examples of WHP activities reported by wellness team members addressed all target behaviors and ranged from discrete annual events to ongoing programs. Centers in New England were more likely to have well-developed programs likely because the regional employee health and safety nurse had initiated and championed these efforts. Centers in Maryland received no customized outreach and reported fewer WHP activities.

Survey response rate was about 72% of the complete workforce rosters of clinical staff members. Nursing aides comprised a majority of respondents (Table 2). Most participants in all facilities were married women aged between 40 and 43 years old. Body mass index (BMI) varied from 27 to 29, placing the average participant in the overweight category (BMI ≥25). This differed by geographic region, as participants in New England were lighter than those in the South, on average.

**Table 2:** Sociodemographic and occupational characteristics of nursing home employees (*n* = 1,589) in 18 skilled nursing facilities classified by level of Worksite Health Promotion (WHP) programing.

**Worksite health promotion programming status Mean** **±** **SD or** ***n*** **(%)**				
**Characteristic (range)**	**Well-developed**	**Emerging**	**No programs**	**Unknown**
	**(2 centers;** ***n*** **=** **226)**^**1**^	**(4 centers;** ***n*** **=** **313)**^**1**^	**(7 centers;** ***n*** **=** **591)**^**1**^	**(5 centers;** ***n*** **=** **459)**^**1**^
**SOCIO-DEMOGRAPHICS**				
Gender^*^				
Female	194 (89.0%)	268 (88.2%)	487 (87.1%)	409 (92.3%)
Male	24 (11.0%)	36 (11.8%)	72 (12.9%)	34 (7.7%)
Race/Ethnicity^*^				
White	184 (81.4%)	158 (50.5%)	240 (41.1%)	191 (41.6%)
Black	15 (6.6%)	73 (23.3%)	262 (44.9%)	229 (49.9%)
Other	27 (11.9%)	82 (26.2%)	82 (14.0%)	39 (8.5%)
**Marital status**				
Married (yes)	128 (57.4%)	157 (50.8%)	289 (49.6%)	223 (49.6%)
Residence^**^				
New England	96 (42.5%)	135 (43.1%)	279 (7.2%)	45 (9.8%)
Maryland	130 (57.5%)	178 (56.9%)	312 (52.3%)	414 (90.2%)
Job category^**^				
Nursing aides	127 (56.2%)	170 (54.3%)	229 (44.0%)	301 (65.6%)
Others	99 (43.8%)	143 (45.7%)	292 (56.0%)	158 (34.4%)
Age (years) (18–78)	42.3 ±12.3	40.2 ±13.0	42.1 ±12.6	42.5 ± 13.1
Education (years) (8–17)	13.4 ± 1.8	13.3 ± 1.9	13.4 ± 1.8	13.3 ± 1.7
**WORKING CONDITIONS**				
Supervisory support (2–8)^*^	5.9 ± 1.3^a^	5.5 ± 1.5	5.5 ± 1.6^ab^	5.5 ± 1.5^b^
Coworker support (2–8)^*^	6.0 ± 1.0	5.7 ± 1.3^a^	5.8 ± 1.2^b^	5.8 ± 1.2
Decision latitude (2–8)^*^	5.1 ± 1.1^ca^	5.2 ± 1.3^b^	5.6 ± 1.2^a^	5.4 ± 1.3
Psychological demand (2–8)	5.7 ± 1.1	5.7 ± 1.0	5.6 ± 1.1	5.7 ± 1.0
Job strain (0–4)	1.2 ± 0.4	1.2 ± 0.5	1.1 ± 0.4	1.1 ± 0.4
Physical exertion (5–20)	12.0 ± 3.5	12.2 ± 3.7	11.6 ± 3.5	12.0 ± 3.5
Safety climate (1–4)	2.8 ± 0.5	2.8 ± 0.5	2.8 ± 0.5	2.8 ± 0.5
Schedule control (2–8)	5.6 ± 1.4	5.6 ± 1.4	5.5 ± 1.4	5.7 ± 1.3
Recent assault at work (yes)	78 (34.4%)	142 (45.4%)	268 (34.4%)	203 (42.2%)

* p < 0.05;

** *p < 0.0001*.

*(1) Number of participants; N's (%) are based on valid responses to survey items. N's vary slightly among the rows, due to missing values*.

*(abc) Letters indicate the mean differences according to Scheffe method*.

Bivariate analyses showed that working conditions were comparable across WHP groups, except for support from supervisors and coworkers as well as decision latitude. Both support constructs were slightly higher in centers with well-developed WHP programs, while decision latitude was lower (Table 2).

A majority of respondents had never smoked, and more than half reported to exercise regularly. The prevalence of not smoking and regular aerobic activity were both slightly lower in centers with well-developed programs (Table 3).

**Table 3:** Health behavior and beliefs outcome characteristics of nursing home employees (*n* = 1,589) in 18 skilled nursing facilities classified by level of Worksite Health Promotion (WHP) programing'.

**Worksite health promotion programming status Mean** **±** **SD or Percentage (%)**				
**Characteristic (range)**	**Well-developed**	**Emerging**	**No programs**	**Unknown**
	**(2 centers;** ***n*** **=** **226)**^**1**^	**(4 centers;** ***n*** **=** **313)**^**1**^	**(7 centers;** ***n*** **=** **591)**^**1**^	**(5 centers;** ***n*** **=** **459)**^**1**^
**Health behavior**				
Smoking (never)	157 (44.6%)	244 (58.0%)	468 (60.7%)	352 (62.3%)
Regular exercise (yes)	118 (52.7%)	177 (57.5%)	333 (57.1%)	272 (59.8%)
Body mass index (12–57)^*^	27.8 ± 5.6	27.9 ± 6.1	29.0 ± 6.5	28.8 ± 6.3
**During the last 3 months have you any of the following (1** **=** **do much less to 5** **=** **do much more often)**				
Eat high fat	2.3 ± 1.1	2.4 ± 1.3	2.4 ± 1.2	2.3 ± 1.2
Eat high fiber	3.3 ± 1.1	3.4 ± 1.2	3.2 ± 1.2	3.3 ± 1.2
Try to lose weight	3.4 ± 1.0	3.4 ± 1.2	3.3 ± 1.2	3.4 ± 1.2
Exercise	3.3 ± 1.1	3.3 ± 1.3	3.2 ± 1.2	3.3 ± 1.2
Have stress in my life^*^	3.1 ± 1.1	3.0 ± 1.2	2.9 ± 1.2	2.9 ± 1.2
Smoke cigarettes/tobacco^2^	2.8 ± 0.9	2.7 ± 1.0	2.8 ± 1.0	2.8 ± 1.0
Drink alcohol	2.6 ± 0.9	2.6 ± 1.1	2.6 ± 1.1	2.6 ± 1.0
Get a full night's sleep^*^	2.8 ± 1.0	2.3 ± 1.1	2.2 ± 1.0^a^	3.1 ± 1.1^b^
**Health perception and beliefs**				
**Health self-efficacy–confidence to do consistently for at least 6 months (1** **=** **not to 4** **=** **very)**				
Avoid eating high fat foods	2.7 ± 0.9	2.8 ± 0.9	2.7 ± 1.0	2.8 ± 1.0
Eat fruits/vegetables	2.9 ± 1.0	2.9 ± 1.0	2.9 ± 1.0	2.9 ± 1.0
Lose or maintain weight^*^	2.6 ± 1.0	2.8 ± 1.0	2.8 ± 1.0^a^	2.9 ± 1.0^b^
Exercise	2.7 ± 1.0	2.8 ± 1.1	2.6 ± 1.0	2.7 ± 1.0
Reduce amount of stress	2.5 ± 1.0	2.6 ± 1.0	2.7 ± 1.0	2.7 ± 1.0
Avoid smoking cigarettes^3^	1.7 ± 0.9	1.9 ± 0.9	1.7 ± 0.9	1.8 ± 0.9
Avoid alcohol or moderate	3.5 ± 0.9	3.5 ± 0.9	3.6 ± 0.9	3.6 ± 0.8
Get a full night's sleep	2.7 ± 1.1	2.7 ± 1.1	2.8 ± 1.0	2.8 ± 1.0
Meet most of job demands	3.4 ± 0.8	3.4 ± 0.8	3.4 ± 0.8	3.5 ± 0.7
**Internal health locus of control**				
IHLOC (6–36)	26.1 ± 5.6	25.1± 6.3	25.5 ± 6.0	26.0 ± 6.4

* *p < 0.05*.

*(1) Number of participants; N's (%) are based on valid responses to survey items. N's vary slightly among the rows, due to missing values for individual questionnaire item*.

*(2) Participants were instructed to answer “no change” if they had been a non-smoker for at least 3 months*.

*(3) Smoking self-efficacy was measured among former and current smokers, which were combined into one category*.

*(ab) Letters indicate the mean differences according to Scheffe method*.

All but one of the health self-efficacy items were similar among programs. Confidence in ability to lose weight or maintain ideal body weight was lowest in centers with well-developed programs. Degree of change in health behavior within last 3 months was similar across program levels, except that participants in centers with well-developed programs reported having slightly more stress and getting slightly less sleep than they used to do (Table 3). Because there were such minimal differences in the health outcomes among groups, there was no power to examine supervisor support as a mediator of the association between facility WHP level and employee health.

After adjusting for workforce socio-demographic characteristics, supervisor support stood out as higher in centers with well-developed programs, compared to centers with no programs (Table 4). Decision latitude was slightly lower in centers with well-developed and emerging programs, compared to those with none. Among behavioral outcomes, BMI was slightly lower among centers with well-developed, emerging, and unknown programs compared with none.

**Table 4:** Adjusted odds ratios from Genmod multivariate regression modeling: One model each for well-developed, emerging, and unknown program status, with no programs as the reference group for each model.

**Dependent**	**Well-developed**	**Emerging**	**Unknown**
**variable program**	**aOR (95% CI)**	**aOR (95% CI)**	**aOR (95% CI)**
Supervisory support	**1.09 (1.02–1.16)**	1.03 (0.96–1.09)	1.03 (0.97–1.10)
Coworker support	1.03 (0.96–1.10)	0.98 (0.92–1.04)	1.00 (0.95–1.06)
Decision latitude	0.94 (0.88–1.01)	**0.94 (1.13–1.00)**	1.00 (0.94–1.06)
Recent increase in having stress in life	1.09 (0.99–1.19)	1.08 (0.99–1.17)	1.04 (0.96–1.13)
Change in getting full night sleep	0.94 (0.86–1.04)	0.98 (0.90–1.07)	1.00 (0.93–1.09)
Lose or maintain ideal weight self–efficacy	0.94 (0.85–1.04)	1.01 (0.93–1.10)	1.01 (0.93–1.10)
Body mass index	**0.95 (0.92–0.98)**	**0.97 (0.94–1.00)**	**0.97 (0.94–0.99)**

*ORs adjusted for worker age, job category, race, gender, and region of residence*.

*adjusted Odds Ratios (aOR) are indicated in bold*.

## Discussion

This non-experimental study examined the association of a company-sponsored WHP in the long-term care sector with workers' health indicators, health beliefs and behaviors, and work environment conditions. There were no major differences across the programs with respect to most outcomes. The prevalence of non-smoking, surprisingly, was lower in the two centers with well-developed programs. Smoking behavior is often established early in life and is notoriously difficult to stop; job stress may be one obstacle to smoking cessation, although the literature is inconsistent (48–50). Failure of smoking cessation programs depends of the type occupational activities for example workers who work during the night are more likely to experience smoking cessation failure, and this could vary by age. Some older workers tend to have more fear about the possibility of health deterioration and some of their symptoms may affect smoking cessation in a positive way (51). Similarly, regular exercise was reported least often by workers in centers with well-developed programs, which may reflect lack of leisure time in this population (39). Among recent behavioral changes, workers in centers with well-developed WHP programs reported experiencing more stress and getting less sleep.

Average BMI was slightly lower in centers with well-developed and emerging WHP programs, even after adjusting for several other health indicators and work environment features. This is in line with literature indicating that higher intensity programs targeting obesity have a better success rate (52). On the other hand, weight self-efficacy was reported less favorably in well-developed programs. BMI reflects a complex mixture of effects of unhealthy diet, lack of aerobic exercise, and stressful life conditions, as well as the “normal” aging process (32). In this same workforce, we have previously demonstrated a linear increase in BMI with number of workplace stressors: poor coworker support, low decision latitude, recent assault(s) at work, work at night, and lifting heavy loads (40). Others have also reported that work factors such as shiftwork play a role in obesity (36). None of these occupational obstacles to weight loss were addressed in the program evaluated here, which may have limited its impact.

In addition to the direct effects of work stressors on health behaviors, job conditions such as psychosocial strain, overtime, and work scheduling also affect participation in health promotion activities in the workplace (53–55). Poor health behaviors have also been associated with low participation in WHP programs, potentially creating a vicious circle (55). Thus, it is salient to consider the contributions of job stressors in this population. Decision latitude was slightly lower in centers with well-developed or emerging WHPs. The WHP activities did not seek to increase decision latitude at work, and there was also no reason to think that they would diminish it. Thus, it is likely that these differences were pre-existing. Low decision latitude could have exerted a small negative confounding effect on the lack of health benefits from facility WHP activities.

There was a weak pattern of higher social support (especially supervisory) where there were well-developed programs compared to none. Supervisory support was not correlated with health behaviors, so there was no indication that it mediated any benefits of the WHP. Social support was not directly targeted by the company's WHP program. However, it may have been the case that centers with more supportive administrators were more likely to implement WHP activities. Issues such as management support, financial resources, and release time for workers to participate in WHP activities have all been identified as potential barriers to a successful WHP program in this same long-term care company (56), so it would not be surprising if there had been self-selection into WHP adoption by more supportive facility administrators.

The findings of our study are in line with the literature with respect to some outcomes and not others. WHP effectiveness is determined by program scope; the most successful programs for positive health and financial outcomes are multi-resourced initiatives, with organizational leadership, health risk screening, individually tailored programs, and a supportive workplace culture (57). In contrast, the current study evaluated a health promotion effort with few resources invested and no WHP professionals to design and implement it. Thus, the limited results are not surprising, and in fact the positive associations, although weak, might be considered unexpectedly encouraging.

In addition to the extent of resources invested by the company, there is a more fundamental possible reason for lack of benefit. Program effectiveness may depend on how much the work environment itself does or does not support healthy behaviors (58). The work of direct healthcare providers is extremely stressful; the interaction between occupational and non-work factors, such as family demands and heath behaviors, could plausibly mitigate against effectiveness of a program that emphasizes individual behavior changes. Reducing those stressors in the work environment could produce “salutogenic” conditions which support rather than interfere with employee health (59). This is the concept underlying the NIOSH *Total Worker Health*®, program (4).

Another potential barrier to participation in WHP programs could be lack of access to group health insurance. In the current study population, a large proportion of nursing aides declined insurance offered by their employer due to its cost. As some of the programs were available via insurance only, not having appropriate coverage could be an obstacle to access.

This study has some important strengths. The response rate was high and similar across the centers, guarding against selection bias. The large population surveyed in different geographical areas was representative of the company workforce (over 200,000 employees). The data on WHP program activities came directly from the representatives responsible for overseeing and/or implementing the programs in each center. The same resources were distributed to all facilities, which all provided similar services, making it possible to evaluate the effect of differential implementation at the center level.

On the other hand, this is a cross-sectional study and, as such, the temporal association between exposure and outcome cannot be determined. The real timing of the advent of the programs is unknown. In addition, details about each program were not complete. We were not able to determine what actual activities were carried out by each center or what was the exact role of the champion, specifically the frequency and intensity of effort devoted to the program and how much this varied among centers. To the best of our knowledge, no center had an on-site fitness program or provided release time for WHP activities, but we could not confirm this with each center.

Five of the 18 nursing centers did not provide the information needed to determine their WHP category. We speculate that these centers classified as missing were most likely to have no WHP programs in place and thus did not respond because they had no information to provide. This would be consistent with the results showing little difference between centers labeled as “no program” and as “unknown.”

## Conclusions

This study's main finding is that a low-intensity, low-resourced workplace health promotion program may have benefited a few individuals but seems to have had only modest influence on average levels of the measured indicators. The fact that the study population was largely low-income women, many with family responsibilities and/or second jobs, may also be partly responsible for the extremely limited benefits observed. Full-time working adults spend more waking hours at work than anywhere else, but a limited program delivery during working hours may not be able to outweigh other influences on their health behaviors. Even if behavioral change is achieved at work, it might not be easily sustainable after work, in part because conditions of employment affect non-occupational factors such as work-family balance, extent of free time, and neighborhood of residence. Like many other WHP programs, this one was designed to help individuals achieve behavioral change without addressing environmental influences. Thus, it is not surprising that it showed limited effect.

Individual behavior is only the top tier of the health pyramid. Worksite programs should be organized as multilevel approaches, accounting for the influence of wages and working conditions, the organizational structure of work (e.g., decision autonomy), the impact of the social environment and work-life balance on health behaviors. They should also be coordinated with other efforts such as community involvement, incentives to the family, or public policy initiatives.

## Data Availability Statement

The data were collected with assurance of confidentiality and privacy to all study subjects, with no provision for data sharing. Requests to access the datasets should be directed to [Laura Punnett, Laura_Punnett@uml.edu].

## Ethics Statement

The studies involving human participants were reviewed and approved by Institutional Review Board University of Massachusetts Lowell. The patients/participants provided their written informed consent to participate in this study.

## Author Contributions

GK conceptualized and carried out the study analyses and drafted the manuscript. MC advised on data analysis and provided critical input on the manuscript. RG supervised data cleaning and management and provided statistical expertise. DK assisted with study conceptualization. LP designed and directed the ProCare study and advised on data analysis and manuscript revisions. All authors contributed to the article and approved the submitted version.

## Conflict of Interest

The authors declare that the research was conducted in the absence of any commercial or financial relationships that could be construed as a potential conflict of interest.

## References

[1] Edington DW Schultz AB Pitts JS Camilleri A. The future of health promotion in the 21st century. Am J Lifestyle Med. (2015) 10:242–52. 10.1177/1559827615605789 30202279 PMC6125058

[2] Occupational Safety and Health Healthy People. (2020). Available online at: https://www.healthypeople.gov/2020/topics-objectives/topic/occupational-safety-and-health (accessed October 10, 2020).

[3] The Henry J Kaiser Family Found. Employer Health Benefits Survey. (2019) Available online at: https://www.kff.org/health-costs/report/2019-employer-health-benefits-survey/019 (accessed October 10, 2020).

[4] Punnett L Henning R Cavallari J Nobrega S Dugan A Cherniack M. Defining “Integration” for total worker health®: a new proposal. Ann Work Exposures Health. (2020) 64:223–35. 10.1093/annweh/wxaa003 32003780 PMC7064271

[5] Jones D Molitor D Reif J. What do workplace wellness programs do? Evidence from the illinois workplace wellness study. Q J Econ. (2019) 134:1747–91. 10.1093/qje/qjz023 31564754 PMC6756192

[6] Pollitz K Rae M Workplace Wellness Programs Characteristics and Requirements. Kaiser Family Foundation. Available online at: https://www.kff.org/private-insurance/issue-brief/workplace-wellness-programs-characteristics-and-requirements/ (accessed January 12, 2020).

[7] Ryan M Erck L McGovern L McCabe K Myers K Nobrega S. “Working on Wellness:” protocol for a worksite health promotion capacity-building program for employers. BMC Public Health. (2019) 19:111. 10.1186/s12889-019-6405-1 30683102 PMC6347764

[8] Baicker K Cutler D Song Z. Workplace wellness programs can generate savings. Health Aff. (2010) 29:304–11. 10.1377/hlthaff.2009.0626 20075081

[9] Pronk NP. Placing workplace wellness in proper context: value beyond money. Prev Chron Dis. (2014) 11:E119. 10.5888/pcd11.140128 25011001 PMC4093978

[10] Berkland BE Werneburg BL Jenkins SM Friend JL Clark MM Rosedahl JK. A worksite wellness intervention: improving happiness, life satisfaction, and gratitude in health care workers. Mayo Clin Proc Innov Qual Outcomes. (2017) 1:203–10. 10.1016/j.mayocpiqo.2017.09.002 30225418 PMC6132199

[11] Biswas A Severin CN Smith PM Steenstra IA Robson LS Amick BC. Larger workplaces, people-oriented culture, and specific industry sectors are associated with co-occurring health protection and wellness activities. Int J Environ Res Public Health. (2018) 15:2739. 10.3390/ijerph15122739 30518161 PMC6313504

[12] Bolnick H Millard F Dugas JP. Medical care savings from workplace wellness programs: what is a realistic savings potential? J Occup Environ Med. (2013) 55:4–9. 10.1097/JOM.0b013e31827db98f 23263453

[13] Claxton G Rae M Damico A Young G McDermott D Whitmore H. Health benefits in 2019: premiums inch higher, employers respond to federal policy. Health Aff. (2019) 38:1752–61. 10.1377/hlthaff.2019.01026 31553631

[14] Kaiser CP Absenteeism, presenteeism, and workplace climate: a taxonomy of employee attendance behaviors. Econ Bus J Inquiries Perspect. (2018) 9:69–86. Available online at: https://pdfs.semanticscholar.org/8ed7/329243e83c3f6fda2074407c0a0d880abcfd.pdf

[15] Edwards A Marcus S Employee perceptions of well-being programs. J Soc Behav Health Sci. (2018) 12100–13. 10.5590/JSBHS.2018.12.1.07

[16] Linnan LA Cluff L Lang JE Penne M Leff MS. Results of the workplace health in America survey. Am J Health Promot AJHP. (2019) 33:652–65. 10.1177/0890117119842047 31007038 PMC6643274

[17] Mattke S Liu HH Caloyeras JP Huang CY Van Busum KR Khodyakov D. Workplace Wellness Programs Study: Final Report. (2013). Available online at: https://www.rand.org/pubs/research_reports/RR254.html (accessed October 10, 2020). 10.7249/rhq-03-02-07 PMC4945172 28083294

[18] Engbers LH van Poppel MNM Chin A Paw MJM van Mechelen W. Worksite health promotion programs with environmental changes: a systematic review. Am J Prev Med. (2005) 29:61–70. 10.1016/j.amepre.2005.03.001 15958254

[19] McCoy K Stinson K Scott K Tenney L Newman LS. Health promotion in small business: a systematic review of factors influencing adoption and effectiveness of worksite wellness programs. J Occup Environ Med. (2014) 56:579–87. 10.1097/JOM.0000000000000171 24905421 PMC4471849

[20] Wierenga D Engbers LH Van Empelen P Duijts S Hildebrandt VH Van Mechelen W. What is actually measured in process evaluations for worksite health promotion programs: a systematic review. BMC Public Health. (2013) 13:1190. 10.1186/1471-2458-13-1190 24341605 PMC3890539

[21] Rongen A Robroek SJW van Lenthe FJ Burdorf A. Workplace health promotion: a meta-analysis of effectiveness. Am J Prev Med. (2013) 44:406–15. 10.1016/j.amepre.2012.12.007 23498108

[22] Stanulewicz N Knox E Narayanasamy M Shivji N Khunti K Blake H. Effectiveness of lifestyle health promotion interventions for nurses: a systematic review. Int J Environ Res Public Health. (2020) 17:17. 10.3390/ijerph17010017 31861367 PMC6981404

[23] Chapman LS. Meta-evaluation of worksite health promotion economic return studies: 2012 update. Am J Health Promot. (2012) 26:TAHP1–12. 10.4278/ajhp.26.4.tahp 22375583

[24] Goetzel RZ Ozminkowski RJ. The health and cost benefits of work site health-promotion programs. Annu Rev Public Health. (2008) 29:303–23. 10.1146/annurev.publhealth.29.020907.090930 18173386

[25] Pelletier KR. A review and analysis of the clinical and cost-effectiveness studies of comprehensive health promotion and disease management programs at the worksite: update VIII 2008 to (2010). J Occup Environ Med. (2011) 53:1310–31. 10.1097/JOM.0b013e3182337748 22015548

[26] Song Z Baicker K. Workplace wellness programs and health outcomes—reply. JAMA. (2019) 322:893. 10.1001/jama.2019.9829 31479133

[27] Horwitz JR Kelly BD DiNardo JE. Wellness incentives in the workplace: cost savings through cost shifting to unhealthy workers. Health Aff Proj Hope. (2013) 32:468–76. 10.1377/hlthaff.2012.0683 23459725

[28] Stiehl E Shivaprakash N Thatcher E Ornelas IJ Kneipp S Baron SL. Worksite health promotion for low-wage workers: a scoping literature review. Am J Health Promot. (2018) 32:359–73. 10.1177/0890117117728607 28893085 PMC5770241

[29] Pomeranz JL Garcia AM Vesprey R Davey A. Variability and limits of US state laws regulating workplace wellness programs. Am J Public Health. (2016) 106:1028–31. 10.2105/AJPH.2016.303144 27077349 PMC4880250

[30] Schwatka NV Smith D Weitzenkamp D Atherly A Dally MJ Brockbank CVS. The impact of worksite wellness programs by size of business: a 3-year longitudinal study of participation, health benefits, absenteeism, and presenteeism. Ann Work Expo Health. (2018) 62 (Suppl. 1):S42–54. 10.1093/annweh/wxy049 30212884 PMC7065409

[31] Cherniack M. Integrated Health Programs, Health Outcomes, and Return on Investment: Measuring Workplace Health Promotion and Integrated Program Effectiveness. J Occup Environ Med. (2013) 55 (12 Suppl):S38–45. 10.1097/JOM.0000000000000044 24284755

[32] Egger G Dixon J. Beyond obesity and lifestyle: a review of 21st century chronic disease determinants. BioMed Res Int. (2014) 2014:731685. 10.1155/2014/731685 24804239 PMC3997940

[33] Healthcare Occupations Occupational Outlook Handbook. U.S. Bureau of Labor Statistics. (2020). Available online at: https://www.bls.gov/ooh/healthcare/home.htm

[34] Konttinen H van Strien T Männistö S Jousilahti P Haukkala A. Depression, emotional eating and long-term weight changes: a population-based prospective study. Int J Behav Nutr Phys Act. (2019) 16:28. 10.1186/s12966-019-0791-8 30894189 PMC6427874

[35] Saulle R Bernardi M Chiarini M Backhaus I La Torre G. Shift work, overweight and obesity in health professionals: a systematic review and meta-analysis. Clin Ter. (2018) 169:e189–97. 10.7417/T.2018.2077 30151553

[36] Sun M Feng W Wang F Li P Li Z Li M. Meta-analysis on shift work and risks of specific obesity types. Obes Rev Off J Int Assoc Study Obes. (2018) 19:28–40. 10.1111/obr.12621 28975706

[37] Qin J Kurowski A Gore R Punnett L. The impact of workplace factors on filing of workers' compensation claims among nursing home workers. BMC Musculoskelet Disord. (2014) 15:29. 10.1186/1471-2474-15-29 24476529 PMC3912896

[38] Miranda H Punnett L Gore RJ ProCare Research Team. Musculoskeletal pain and reported workplace assault: a prospective study of clinical staff in nursing homes. Hum Factors. (2014) 56:215–27. 10.1177/0018720813508778 24669555 PMC11837844

[39] Zhang Y Punnett L Mawn B Gore R. Working conditions and mental health of nursing staff in nursing homes. Issues Ment Health Nurs. (2016) 37:485–92. 10.3109/01612840.2016.1162884 27104634 PMC5886762

[40] Miranda H Gore RJ Boyer J Nobrega S Punnett L. Health behaviors and overweight in nursing home employees: contribution of workplace stressors and implications for worksite health promotion. Sci World J. (2015) 2015:915359. 10.1155/2015/915359 26380373 PMC4561990

[41] Ware J Kosinski M Keller SD. A 12-Item short-form health survey: construction of scales and preliminary tests of reliability and validity. Med Care. (1996) 34:220–33. 10.1097/00005650-199603000-00003 8628042

[42] Karasek R Brisson C Kawakami N Houtman I Bongers P Amick B. The job content questionnaire (JCQ): an instrument for internationally comparative assessments of psychosocial job characteristics. J Occup Health Psychol. (1998) 3:322–55. 10.1037/1076-8998.3.4.322 9805280

[43] Büssing A Social tolerance of working time scheduling in nursing. Work Stress. (1996) 10:238–50. 10.1080/02678379608256803

[44] Griffin MA Neal A. Perceptions of safety at work: a framework for linking safety climate to safety performance, knowledge, and motivation. J Occup Health Psychol. (2000) 5:347–58. 10.1037/1076-8998.5.3.347 10912498

[45] Bandura A Self-efficacy: The exercise of control. W H Freeman/Times Books/Henry Holt & Co. (1997).

[46] Lee S Lee DK. What is the proper way to apply the multiple comparison test? Korean J Anesthesiol. (2018) 71:353–60. 10.4097/kja.d.18.00242 30157585 PMC6193594

[47] Greenland S. Modeling and variable selection in epidemiologic analysis. Am J Public Health. (1989) 79:340–9. 10.2105/AJPH.79.3.340 2916724 PMC1349563

[48] Albertsen K Borg V Oldenburg B. A systematic review of the impact of work environment on smoking cessation, relapse and amount smoked. Prev Med. (2006) 43:291–305. 10.1016/j.ypmed.2006.05.001 16787657

[49] Heikkilä K Nyberg ST Fransson EI Alfredsson L Bacquer DD Bjorner JB. job strain and tobacco smoking: an individual-participant data meta-analysis of 166 130 adults in 15 European studies. PLoS ONE. (2012) 7:e35463. 10.1371/journal.pone.0035463 22792154 PMC3391192

[50] Liu J Zhao S Chen X Falk E Albarracín D. The influence of peer behavior as a function of social and cultural closeness: a meta-analysis of normative influence on adolescent smoking initiation and continuation. Psychol Bull. (2017) 143:1082–115. 10.1037/bul0000113 28771020 PMC5789806

[51] Cho YM Kim HR Kang M-Y Myong J-P Koo JW. Fixed night workers and failed smoking cessation. J Occup Med Toxicol Lond Engl. (2019) 14:23. 10.1186/s12995-019-0243-z 31404360 PMC6683487

[52] Tabak R Hipp JA Dodson EA Yang L Adlakha D Brownson RC. Exploring associations between perceived home and work neighborhood environments, diet behaviors, and obesity: results from a survey of employed adults in missouri. Prev Med Rep. (2016) 4:591–6. 10.1016/j.pmedr.2016.10.008 27843759 PMC5107640

[53] Ghesmaty Sangachin M Cavuoto LA. Interactive effects of work psychosocial factors on participation in workplace wellness programs. J Workplace Behav Health. (2018) 33:24–42. 10.1080/15555240.2017.1408415 29599663 PMC5868489

[54] Hunter JR Gordon BA Bird SR Benson AC Perceived barriers and facilitators to workplace exercise participation. Int J Workplace Health Manag. (2018) 11:349–63. 10.1108/IJWHM-04-2018-0055

[55] Jørgensen MB Villadsen E Burr H Punnett L Holtermann A. Does employee participation in workplace health promotion depend on the working environment? A cross-sectional study of danish workers. BMJ Open. (2016) 6:e010516. 10.1136/bmjopen-2015-010516 27279474 PMC4908961

[56] Zhang Y Punnett L Nannini A. Work-family conflict, sleep, and mental health of nursing assistants working in nursing homes. Workplace Health Saf . (2017) 65:295–303. 10.1177/2165079916665397 27794076 PMC8556705

[57] Hendriksen IJM Snoijer M de Kok BPH van Vilsteren J Hofstetter H. Effectiveness of a multilevel workplace health promotion program on vitality, health, and work-related outcomes. J Occup Environ Med. (2016) 58:575–83. 10.1097/JOM.0000000000000747 27136605 PMC4883645

[58] Nobrega S Champagne N Abreu M Goldstein-Gelb M Montano M Lopez I. Obesity/overweight and the role of working conditions: a qualitative, participatory investigation. Health Promot Pract. (2016) 17:127–36. 10.1177/1524839915602439 26333770 PMC5860907

[59] Jenny GJ Bauer GF Vinje HF Vogt K Torp S The application of salutogenesis to work. In: Mittelmark MB, Sagy S, Eriksson M, Bauer MF, Pelikan JM, Lindström B, Espnes GA, editors. The Handbook of Salutogenesis. Cham: Springer (2017). 10.1007/978-3-319-04600-6_20 28590629

